# Supplementary figures and images for: An Updated Staging System for Cephalochordate Development: One Table Suits Them All
Source: Front Cell Dev Biol. 2021 May 20;9:668006. doi: 10.3389/fcell.2021.668006 (PMC8174843; doi:10.3389/fcell.2021.668006)

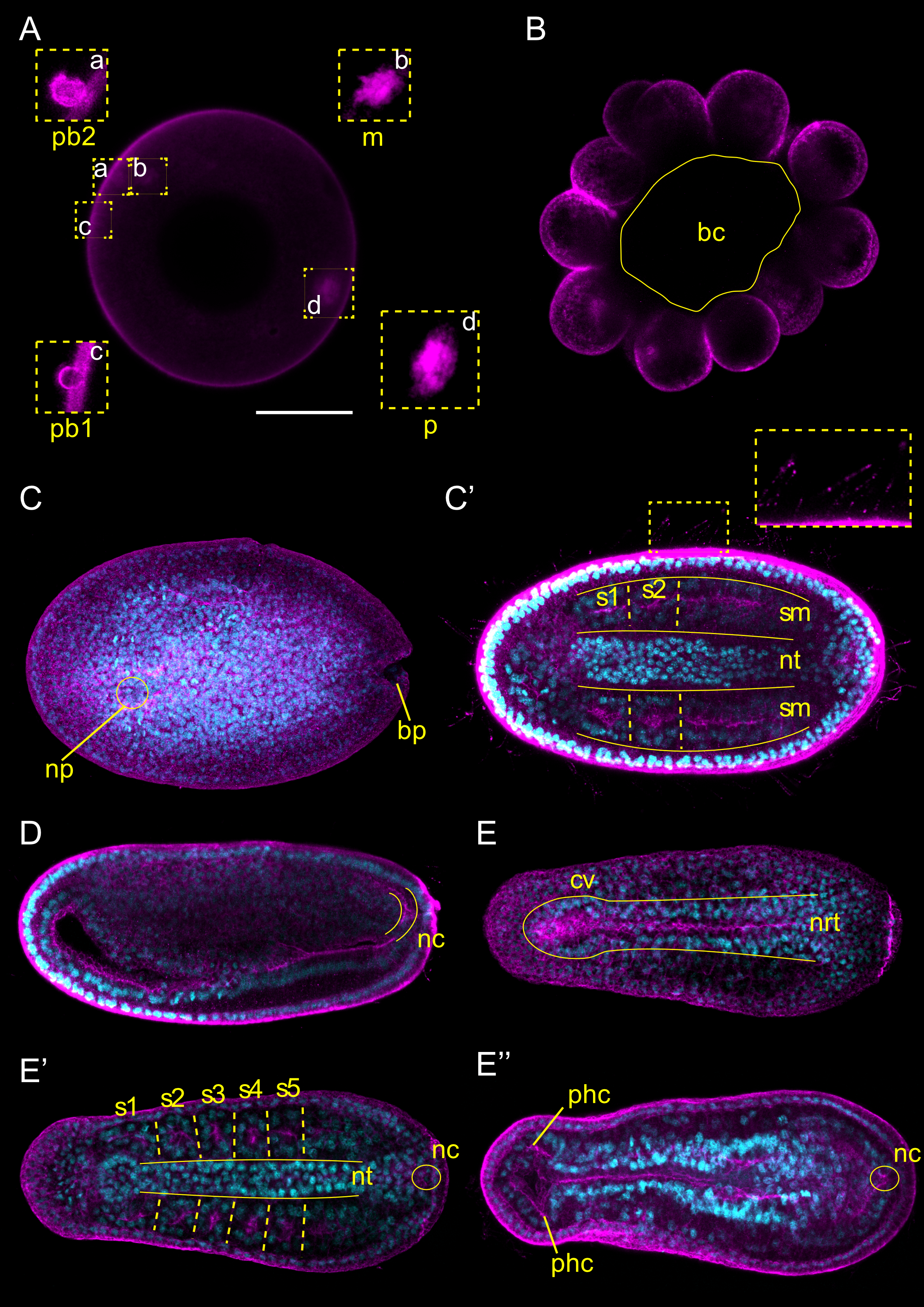

Supplement: Supplementary Figure 1 — Detailed highlights of specific structures of Branchiostoma lanceolatum development during cleavage and neurula stages. (A,B) Embryos are stained with the lipophilic dye FM 4-64 (magenta). (C–E) Embryos are labeled for aPKC (magenta) and stained with the DNA dye Hoechst (cyan). The anterior pole is to the left, and, on the dorsal views, the right side is up (C,C′,E–E″), while, on the lateral view, the dorsal side is up (D). Maximum projections of confocal z-stacks of B. lanceolatum embryos at the 1 cell-stage (A), 32-cell stage (B), N1 stage (C,C′), and N2 stage (D–E″). Insets (a–d) in (A) correspond to regions highlighted with dotted rectangles and are shown at 2x magnification. bc, blastocoel; bp, blastopore; cv, cerebral vesicle; m, maternal DNA; nc, neurenteric canal; np, neuropore; nrt, neural tube; nt, notochord; p, paternal DNA; pb1, 1st polar body; pb2, 2nd polar body; phc, presumptive head cavities; s1–5, somite pairs 1 to 5; sm, somitic mesoderm. Scale bar: 50 μm. [file Image_1.tiff]

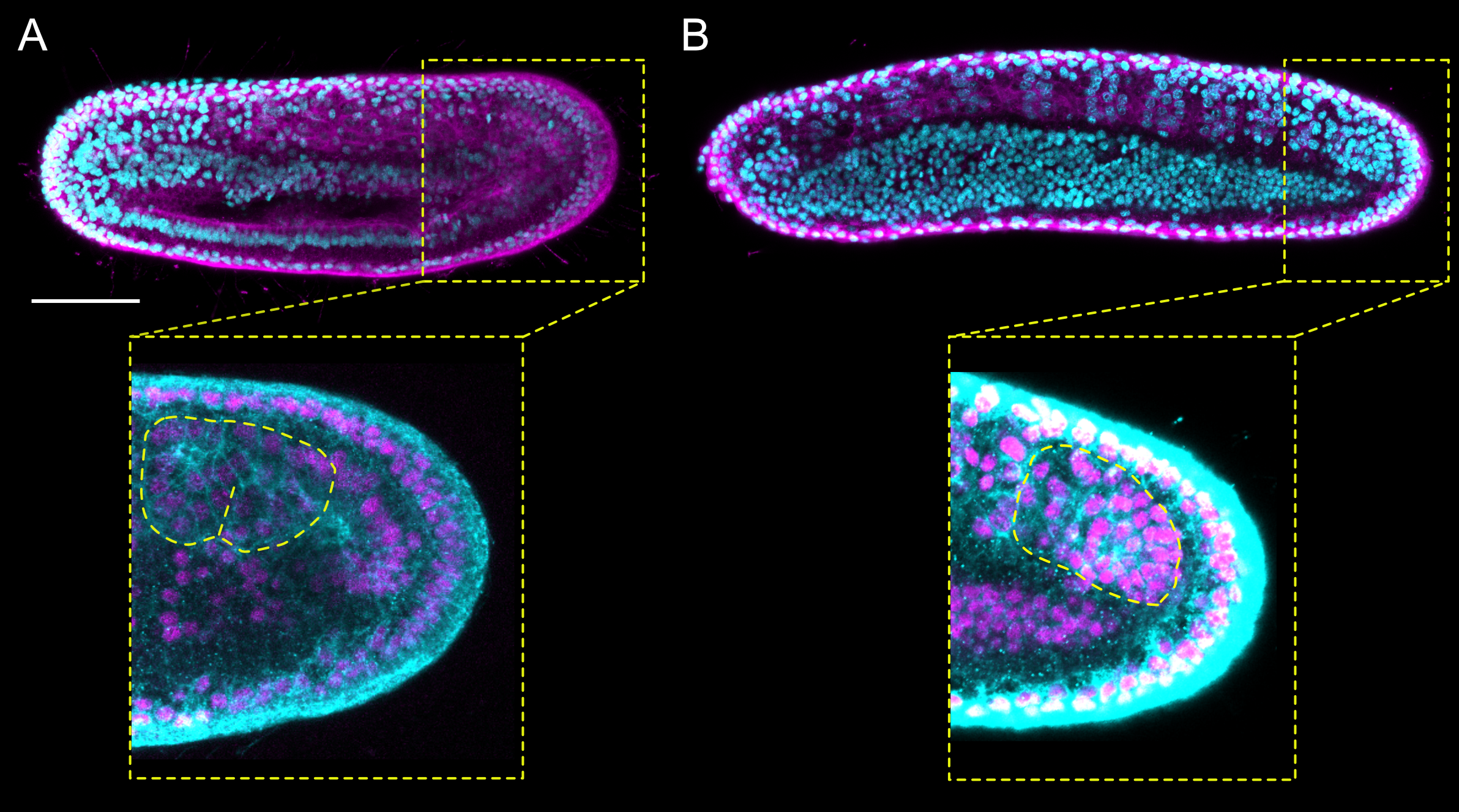

Supplement: Supplementary Figure 2 — Detailed highlights of specific structures of Branchiostoma lanceolatum development during neurula stages. (A,B) Embryos are labeled for aPKC (magenta) and stained with the DNA dye Hoechst (cyan), with the colors having been inverted in the insets for clarity. Insets correspond to regions highlighted with dotted rectangles and are shown at 2x magnification. The anterior pole is to the left, and the dorsal side is up. Single z-stacks show the formation of a new somite by enterocoely at the N4 stage (A) and by schizocoely from the tail bud at the N5 stage (B). Scale bar: 50 μm. [file Image_2.tiff]

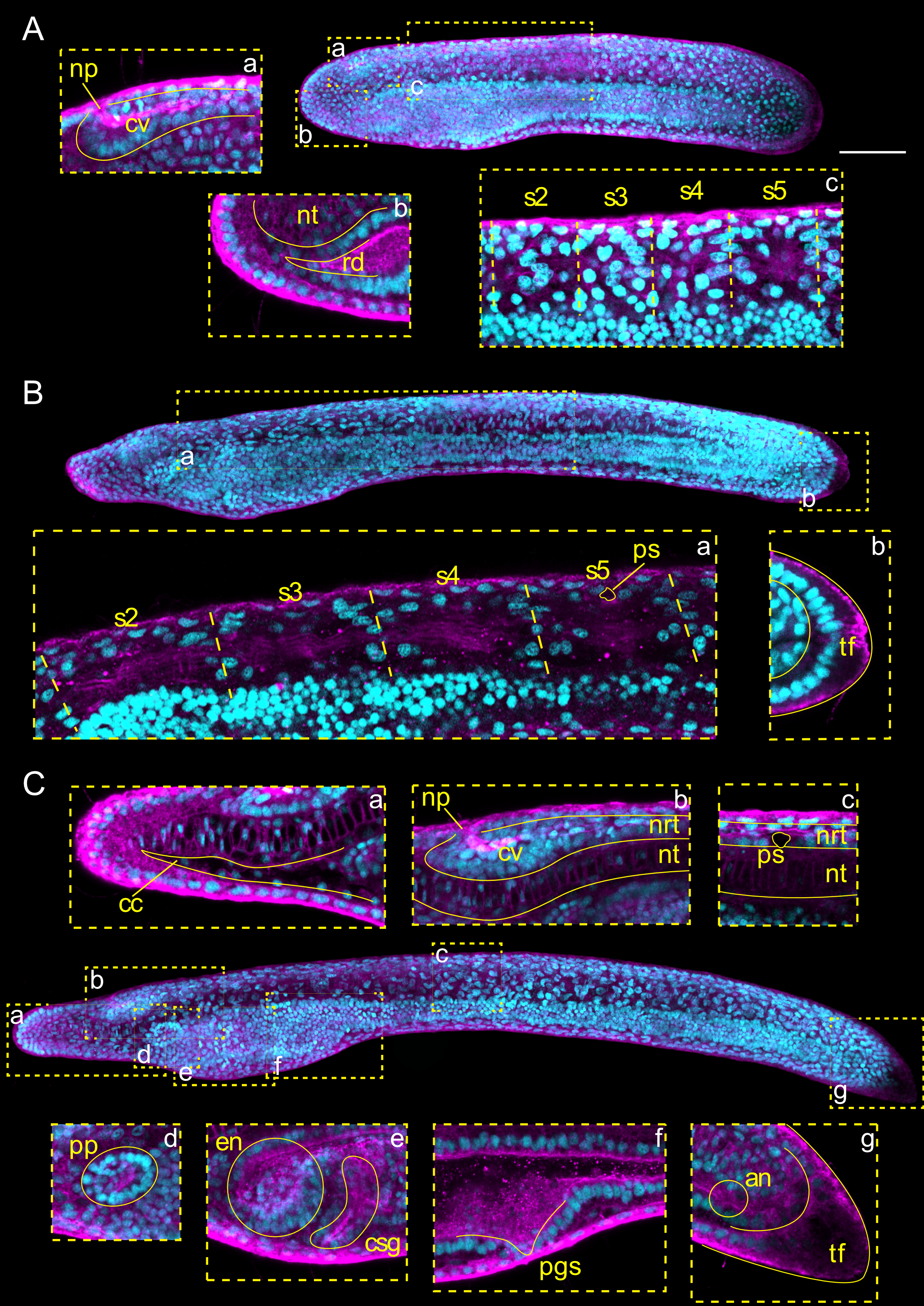

Supplement: Supplementary Figure 3 — Detailed highlights of specific structures of Branchiostoma lanceolatum development during tailbud and larval stages. Embryos and larvae are labeled for aPKC (magenta) and stained with the DNA dye Hoechst (cyan). Embryos and larvae are in lateral views, the anterior pole is to the left and the dorsal side is up. T0 (A), T1 (B), and L0 (C) stages are shown. Insets (a–g) in (C) correspond to regions highlighted with dotted rectangles and are shown at 2x magnification. an, anus; cc, cephalic coelom; csg, club-shaped gland; cv, cerebral vesicle; en, endostyle; np, neuropore; nrt, neural tube; nt, notochord; pgs, presumptive 1st gill slit; pp, pre-oral pit; ps, 1st pigment spot; rd, right diverticulum; s2–5, somite pairs 2 to 5; tf, tail fin. Scale bar: 50 μm. [file Image_3.tiff]

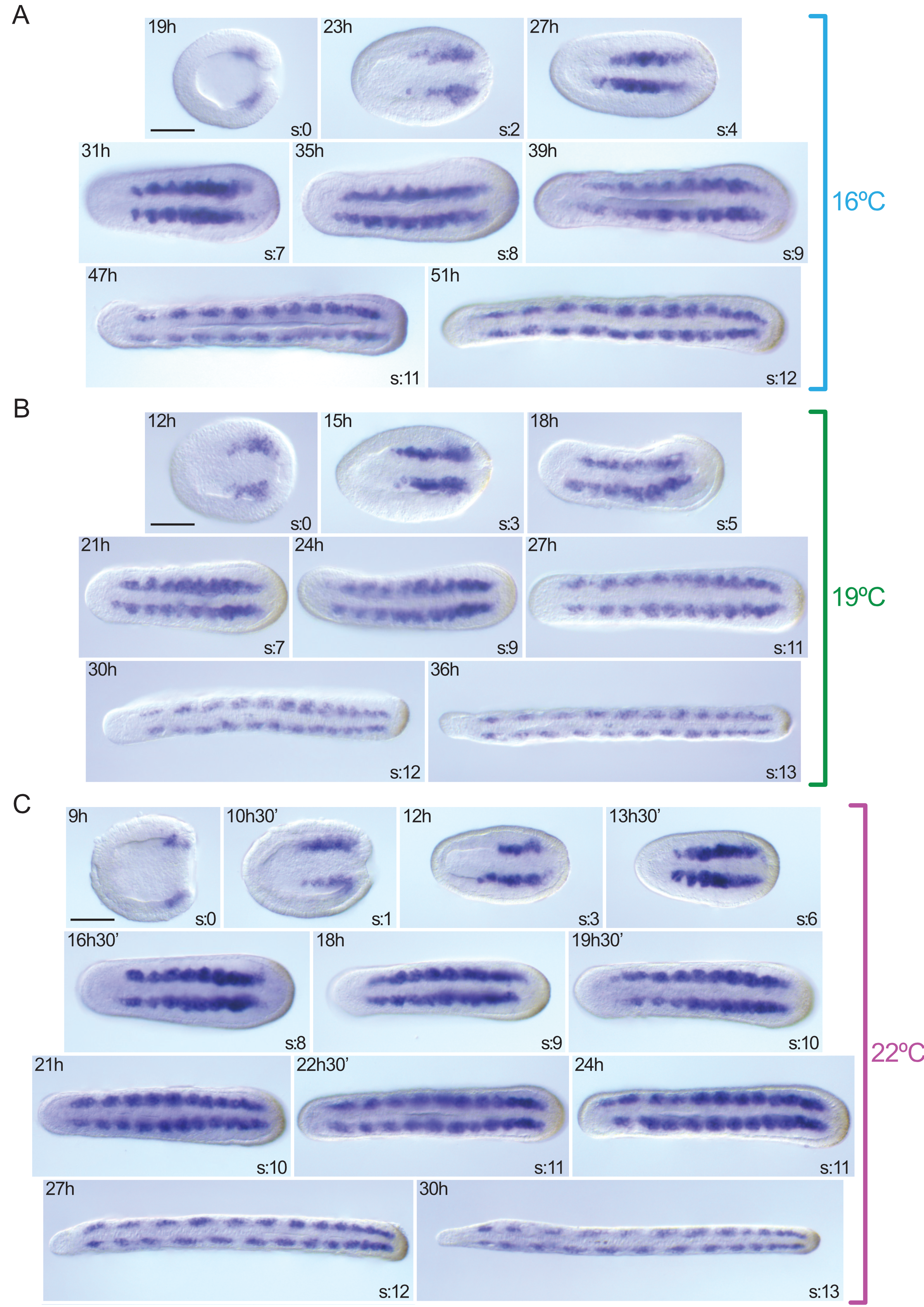

Supplement: Supplementary Figure 4 — Expression of the mrf1 gene in developing Branchiostoma lanceolatum reared at different temperatures. Embryos are in dorsal views with anterior pole to the left and right side up. (A) 16°C, (B) 19°C, and (C) 21°C. On each image, the time of development in hours post fertilization (h) and the number of fully formed somite pairs (s) are indicated. Scale bars: 50 μm. [file Image_4.tiff]
